# Elements for harnessing participatory action research to strengthen health managers’ capacity: a critical interpretative synthesis

**DOI:** 10.1186/s12961-018-0306-0

**Published:** 2018-04-19

**Authors:** Moses Tetui, Joseph Mumba Zulu, Anna-Karin Hurtig, Elizabeth Ekirapa-Kiracho, Suzanne N. Kiwanuka, Anna-Britt Coe

**Affiliations:** 1 Makerere University College of Health Sciences, School of Public Health (MakCHS-SPH), New Mulago Complex, P.O. B0X 7072, Kampala, Uganda; 2 Epidemiology and Global Health Unit, Department of Public Health and Clinical Medicine, Umeå University, 901 87 Umeå, Sweden; 3 School of Public Health, University of Zambia, P.O. Box 50110, Lusaka, Zambia; 4 Sociology Department, Umeå University, 901 87 Umeå, Sweden

**Keywords:** Participatory Action Research, factors, harnessing, health managers’ capacity, systems thinking, implementation research

## Abstract

**Background:**

Health managers play a key role in ensuring that health services are responsive to the needs of the population. Participatory action research (PAR) is one of the approaches that have been used to strengthen managers’ capacity. However, collated knowledge on elements for harnessing PAR to strengthen managers’ capacity is missing. This paper bridges this gap by reviewing existing literature on the subject matter.

**Methods:**

A critical interpretive synthesis method was used to interrogate eight selected articles. These articles reported the use of PAR to strengthen health managers’ capacity. The critical interpretive synthesis method’s approach to analysis guided the synthesis. Here, the authors interpretively made connections and linkages between different elements identified in the literature. Finally, the Atun et al. (Heal Pol Plann, 25:104–111, 2010) framework on integration was used to model the elements synthesised in the literature into five main domains.

**Results:**

Five elements with intricate bi-directional interactions were identified in the literature reviewed. These included a shared purpose, skilled facilitation and psychological safety, activity integration into organisational procedures, organisational support, and external supportive monitoring. A shared purpose of the managers’ capacity strengthening initiative created commitment and motivation to learn. This purpose was built upon a set of facilitation skills that included promoting participation, self-efficacy and reflection, thereby creating a safe psychological space within which the managers interacted and learnt from each other and their actions. Additionally, an integrated intervention strengthened local capacity and harnessed organisational support for learning. Finally, supportive monitoring from external partners, such as researchers, ensured quality, building of local capacity and professional safety networks essential for continued learning.

**Conclusions:**

The five elements identified in this synthesis provide a basis upon which the use of PAR can be harnessed, not only to strengthen health managers’ capacity, but also to foster other health systems strengthening initiatives involving implementation research. In addition, the findings demonstrated the intricate and complex relations between the elements, which further affirms the need for a systems thinking approach to tackling health systems challenges.

## Background

A health system can be described as being comprised of six key components, the health workforce, medical products and technologies, service delivery, information and research systems, financing mechanisms, and leadership and governance, with people at its centre [1]. Health systems around the world face several and differing challenges in delivering quality services to the population. In low-income countries, such challenges include low financing, few and poorly motivated health workers, inaccurate and incomplete records, a persistent shortage of drugs, supplies and needed medical technologies, and a low political will at leadership and governance levels to improve people’s health [2, 3]. Additionally, weak health managers’ capacity exacerbates these challenges [4, 5].

Strengthening managers’ capacity to efficiently and effectively utilise available resources can make systems more responsive [6]. Management is the process by which different resources are organised to achieve a set goal, in this case, the sustainable provision of quality health services to the population [7]. Health management could broker human relations and linkages within and between the different components of the health system [8, 9]. Health managers, through their various actions, have the potential to promote linkages amongst the different components of the health system, thus creating synergy among them [7]. For example, by undertaking planning and budgeting, they interface with the political leadership, the finance departments and product suppliers in a collaborative manner [10]. Therefore, to improve health systems’ responsiveness, there is a need to strengthen health managers’ capacity [6, 11].

To carry out their functions [6], managers require skills that allow them to collaborate with different stakeholders, control the resources they hold and creatively utilise the resources they have while generating more to achieve set goals in a responsive manner [7, 12]. These functions are typically complementary and performed in a simultaneous manner, which makes management complex [7]. Additionally, the increasingly multifaceted environment within which health systems in the 21st century operate exacerbates this complexity [7, 13]. Managers must therefore not only be pragmatic, but also highly dynamic, or in other words, behaviourally complex [7].

Participatory action research (PAR), sometimes referred to as the conceptually similar term ‘action learning’ (the term PAR is used herein for the sake of consistency), is among the many approaches used to strengthen managers’ capacity. Others include formal academic training, institutional experiences and specific short-term workshops [14]. While all of these approaches have unique strengths and weaknesses [6, 14], our focus in this paper is on PAR [15].

We define PAR as an iterative approach to research or learning that actively involves the populations being researched as agents of change [16]. To overcome social challenges, the approach works through the core principles of free and open participation, flexibility, collaboration, theory testing, reflexivity and learning. PAR happens within a cyclic pattern that involves collective identification of a problem, finding the most suitable solution, implementing the solution, monitoring and evaluation, and learning [16].

PAR has long been used to strengthen managers’ capacity in both business and healthcare organisations [17]. In high-income countries, the approach has been utilised since the 1980s to build health managers’ capacity [18]. However, in low-income settings, while the use of PAR to strengthen health managers’ capacity is increasing, most documented knowledge on its use is in community empowerment interventions [18, 19]. Studies show that health managers’ capacity is achieved by actively involving managers in questioning and resolving day-to-day dynamic and complex management challenges. In so doing, managers master learning skills relevant for solving dynamic and complex management challenges [20, 21].

The challenges of using PAR have also been cited, including triggering conflict among participants, time intensity, ambiguity and being overly demanding [22]. Nonetheless, some of these challenges are viewed as necessary for change to occur. For example, well-managed conflict is widely viewed as important for generating organisational change because it triggers alternative thinking and creativity [23].

Despite extensive knowledge of the benefits and challenges of PAR for strengthening health managers’ capacity, there is much less clarity about the collated elements required for harnessing PAR in practice. The aim of our study was therefore to explore the elements for the harnessing of PAR to strengthen health managers’ capacity.

In addition, the paper contributes knowledge on the use of PAR to strengthening health managers’ capacity given its increasing importance to health systems strengthening and implementation research in recent times [16, 23]. The use of flexible approaches when undertaking implementation research has long been advocated for [24, 25]. Flexible approaches resonate with the systems thinking ideology by permitting the accommodation of multiple stakeholders, which creates room for dialogue, reflection and continuous learning [16, 26]. This has recently been re-emphasised by the well-known Health Systems Global network through its thematic working groups as well as by a recently launched online portal to promote the use of PAR within health systems research [16, 27].

In order to investigate these elements, we conducted a Critical Interpretive Synthesis (CIS) of published studies on the topic [16]. From our literature search, no such synthesis had been undertaken and published at the time  (September to December 2016).

## Methods

### Study design

The motivation of this synthesis emerged from findings of an earlier study conducted by the same research team [28]. The previous study explored stakeholder experiences of using a PAR approach to strengthen local health systems. Although findings from this study demonstrated how the PAR approach was experienced as a potential approach for strengthening local health systems, the question of what elements were required to harness PAR remained unanswered. This synthesis was designed to fill this gap by addressing the question, what are the required elements for harnessing PAR to strengthen health managers’ capacity? To examine this question, we drew upon existing literature and knowledge on the topic. Specifically, we used the CIS method of literature review and synthesis [29].

The CIS method was selected for three reasons. Firstly, CIS is a systematic method that facilitates the analysis of complex and diverse bodies of literature with a particular strength in the synthesis of qualitative literature [30]. The available literature on PAR is largely qualitative, making the CIS method relevant for this paper since it permitted the use of qualitative principles [29, 31, 32]. In addition, CIS allows the development of new concepts and theories through an interpretive mode of inquiry, which fit with the aim of our review, namely the exploration of elements for harnessing PAR to strengthen health managers’ capacity. Finally, CIS offered a more “flexible, iterative, dynamic, and reflective approach”, which enabled the assessment of the extent to which new information or data on PAR are provided with each additional paper that was considered in this review [29, 30]. The process of conducting the CIS included conducting a literature search, determining eligibility criteria, quality appraisal, and data extraction and analysis. In the analysis process, three ways of interrogating the literature are provided in the CIS method depending on the research question, these are reciprocal translational analysis, refutational synthesis and the lines of argument (LOA) synthesis. Our research question was best suited for the LOA analysis approach, which involves a constant comparison of different accounts to develop concepts of meaning [29]. Below, we provide more details on these methodological issues.

### Literature search

Following the CIS method, we generally searched electronic databases, undertook reference chaining, and contacted experts in PAR and health management for references. In searching electronic databases, we used general search terms such as PAR, Action Learning, management, capacity-building and health, both independently and in combination. The search did not specifically target studies that focused directly on elements for harnessing PAR to strengthen managers’ capacity as would be in classical systematic reviews. Rather, a broad and flexible search was adopted to include such studies but also others with potentially relevant information [29, 30]. Table 1 shows the literature search steps undertaken from the different sources. The search within the electronic databases and reference chaining was independently undertaken by two of the authors (MT and JMZ). In addition, MT contacted two experts for references as a final step in the literature search process. A review of the list of papers identified was then undertaken and discussed among all the authors in an iterative manner, yielding the papers that were finally included in the review.

**Table 1:** Literature search and selection process

Search words used independently and in combination	Participatory action research, action learning, management, health managers, capacity-building, management strengthening, action research			
Database	Hint records	Number selected after review of title	Number selected after review of abstract	Number selected after review of article
PubMed	3048	10	6	3
Science direct	20,576	27	15	2
Biomed Central	1183	30	5	0
Reference chaining		20	5	2
Expert references		10	4	1
Total number	24,807	97	35	8

The inclusion criteria for the selection of papers to review were that (1) the papers had to have been published in peer-reviewed journals, which also served as a quality check, (2) the paper’s focus had to be the use of PAR to improve health managers’ capacity, and (3) the use of PAR in the papers had to have occurred in real work settings (districts, hospitals, health facilities, etc.) and not in learning or training institutions. With these criteria, eight papers were included in the review.

Finally, the eight included papers were subjected to a quality assessment. We adopted the two-pronged approach of assessing quality as advocated by Dixon and Woods [29]. In this approach, studies that are fatally flawed should be excluded. To identify such studies, we used the criteria proposed by the National Health Service (NHS) National Electronic Library for Health for the assessment of qualitative research [33], answering the five questions listed, namely (1) are the aims and objectives of the research clearly stated? (2) Is the research design clearly specified and appropriate for the aims and objectives of the research? (3) Do the researchers provide a clear account of the procedure by which their findings were produced? (4) Do the researchers display enough data to support their interpretations and conclusions? (5) Is the method of analysis appropriate and adequately explicated?

Secondly, while undertaking the synthesis, we reflected on the credibility and contribution of each of the selected papers in accordance with the CIS approach. All eight papers met the quality assessment criteria. However, three of the studies [19, 34, 35] did not have an explicit explanation of the data analysis process. Since they were relevant for the review, we decided to include them because relevance is a key consideration for inclusion in this kind of interpretive review. A brief description of the papers reviewed is given in Table 2.

**Table 2:** A brief description of papers reviewed

No	Authors, year and country	Population targeted	Description of the intervention	Methods
1	Authors: Louise Doyle [19] Year: 2014 Country: Ireland	Health Managers	The Leaders Edge project. To enhance the capacity of managers to bring about change and increase their exposure to wider organisational issues and challenges. Each participant identified a project that they would undertake, that was of interest to them and of importance to the organisation. The process involved working in small groups to tackle important organisational issues or problems and learn from their attempts to change things. Project was conducted between November 2012 and May 2013	Data collected through meeting review minutes, feedback from participants, an anonymous questionnaire and facilitator reflections/observations. Approach to analysis not specified.
2	Authors: Elizabeth Barnett, Sydney Ndeki [35] Year:1992 Country: Tanzania	District Health Management Team (DHMT)	The project was named district action research and education (DARE). To improve district health management using action research. It was aimed at supporting management by action-oriented problem solving. The strategy involved the DHMT in an iterative process of problem analysis, action research, problem solving and review. The process further combined start-up and review workshops with on-going work by the DHMT to tackle problems of primary healthcare. The project took 12 months (actual dates not specified).	Data collected through meeting minutes, workshop reports, participant feedback and facilitator reflections/observations. Approach to analysis not specified
3	Authors: Catherine Blanchard, Bryan Carpenter [41] Year: 2012 Country: KwaZulu-Natal, South Africa	Health facility managers and higher-level managers	Piloting an action learning group programme with managers in a rural public health setting and to explore participants’ experience of the action-learning programme. This happened in rural KwaZulu-Natal, South Africa. Project took 11 months (actual dates not specified).	Data collected through focus group discussions. Thematic analysis was undertaken in an iterative manner.
4	Authors: Sandra G Leggat, Cathy Balding, Julie Anne Anderson [18] Year: 2011 Country: Australia	Healthcare managers	Training programme using action-learning sets designed to enhance the management abilities of healthcare managers. The programme aimed at equipping the participants with a range of strategies to act on challenges of organisational change. It explored the team psychological safety during the PAR process. In addition, it explored the impact of action learning on empowerment and self-efficacy as key management constructs. Project implemented for 1 year 2007–2008.	Data was collected through a baseline questionnaire before the initiation of PAR and an evaluative questionnaire at the end. The analysis process measured the team psychological process using a 5-point Likert scale on psychological safety. For self-efficacy, a 10-item general perceived efficacy scale was used. For psychological empowerment, the Spreitzer 12-item scale was used. ANVOA and *t* tests were used accordingly for analysis.
5	Authors: Comfort Mshelia, Gillian Le, Tolib Mirzoev Samuel Amon, Ambrose Kessy, Sebastian Olikira Baine, Reinhard Huss [38] Year: 2016 Countries: Ghana, Tanzania and Uganda	DHMT	PERFORM project. Aimed at improving health management capacity using action research. This paper focused on how diaries help action research participants to be more reflective which is a central aspect of action learning. The study was undertaken in Tanzania, Ghana and Uganda between 2011 and 2015. The guidelines emphasised that the DHMTs were free to adapt the formats for their diaries but provided instructions on content.	Data collection was undertaken through review of on-going project documents such as meeting minutes, field visit reports and a PAR handbook developed by researchers. In addition, semi-structured interviews and peer-reviewed literature review was undertaken. An iterative thematic analysis process was undertaken
6	Authors: Cynthia Roberts, David Coghla [39] Year: 2011 Country: USA	Middle level health managers, supervisors, team leaders, drawn from caregivers, diagnostics, administrative support, ancillary services and off-site satellite facilities	LEAD (leadership education and development) institute. A leadership development programme using action research in Midwest USA. Aim is to improve leadership at different levels using action learning through concentric collaborations. Project duration 3 years (actual years not specified).	Data was collected through anonymous feedback notes from workshop participants, individual and collaborative reflection notes, focus group discussions (confidential lunch time meetings), one-on-one dialogue notes with participants and facilitator observations/reflections. Iterative thematic analysis.
7	Authors: Sarah Young, Denise Hinge, Jan Mcfadyen, Vanessa Wright, Pauline Lambert, Carolyn Pilkington, Christine Newsome [34] Year: 2010 Country: UK	Nurse consultants in managerial positions	A facilitated action learning set aimed at supporting the strategic leadership development of eight nurse consultant posts across two National Health Service Trusts in the UK. Project duration 3 years (actual years not specified).	Data was collected through feedback notes at learning sessions. Facilitator observations/reflections. Analysis approach not specified.
8	Authors: Martin S McNamara, Gerard M Fealy, Mary Casey, Tom O’Connor, Declan Patton, Louise Doyle, Christina Quinlan [40] Year: 2013 Country: Ireland	Nurses and midwives in managerial positions	The pathway leadership development intervention. To assess the mentoring, coaching and action learning interventions used to develop nurses’ and midwives’ clinical leadership competencies and to describe the programme participants’ experiences of the interventions in Ireland. This took place from July to December 2011.	Data was collected through focus groups, individual interviews and observations. Analysis was undertaken through an iterative thematic content approach.

### Interrogating the literature

Two of the authors (MT and JMZ) led the data analysis. Following the LOA approach to analysis, they each independently reviewed the papers that were identified as relevant for the review. This stage was meant to allow familiarisation with the existing literature. Next, MT and JMZ undertook a more focused review in which they identified the elements  responsible for the success of the PAR projects aimed at improving managers’ capacity. MT and JMZ shared and discussed the identified elements with each another, which yielded agreement on relevant concepts or constructs identified in the papers reviewed.

MT and JMZ then shared the preliminary concepts with the rest of authors for review and discussion. After reaching agreement on the relevant concepts or constructs, the analysis moved to the next level. MT and JMZ continued the analysis by separately grouping and regrouping the list of concepts or synthetic constructs. Synthetic constructs refer to the meanings derived from critically and interpretively examining the literature according to the CIS method [29]. In accordance to the LOA approach, grouping of the constructs was undertaken, which involved an iterative process of seeking linkages and relationships between the synthetic constructs. This process yielded an interpretation of the linkages and relationships to form synthesising arguments that attempted to explain the relations among the constructs [29, 36].

The arguments were then analysed through the lens of the Atun et al. [37] framework on integration of health interventions to form the five final domains of the synthesis. This conceptual framework identifies domains useful for the successful integration of health interventions into existing health systems [37]. According to this framework, five domains, namely the ‘problem’ being addressed, the ‘intervention’, the ‘adoption system’, the ‘health system characteristics’, and the ‘broad context’, interact in bi-directional ways to influence the successful application of an intervention. We adopted the framework to explore the elements for harnessing PAR to strengthen health managers’ capacity. The framework further allowed us to interpret the bi-directional relationships between domains that became apparent in our synthesis of the literature. Table 3 demonstrates the movement from synthetic constructs to the main domains of the synthesis. Finally, five interrelated domains are presented and discussed in detail in the results.

**Table 3:** Steps of the analysis process

Synthetic constructs	Number of papers	Synthesising argument	Domain
Involved, individual interest, motivation, common gaps, common purpose	7	A shared purpose Individual motivation Focus on individual managers’ capacity development	Problem: A shared purpose
Stretching of participants’ capacity, focus on participants’ unique needs, mix of participants, cultivate trust in groups, encouraging group bounding, promote new insights, promote questioning and new insights, reflection, confidence, demystification of myths Open discussions, open and free learning, supporting each other, linkages and networks, learning within groups, no fear	8	Tailored facilitation, group management, pragmatism, meeting preparations, reflective thinking, promoting self-efficacy Non-threatening environment, social capital, heterogonous learning groups, confidentiality	Intervention: Skilled facilitation and social psychological safety
Locally coordinated, local monitoring, ensure regular attendance, documentation, stability, managers competing demands, sustaining PAR processes, sustaining learning, in sync with usual work Local ownership, use of local operational procedures, mainstreaming, usual duties, managing project demands, overburdened health managers, documentation demands	6	Local champions Local monitoring Sustaining learning Quality control Integration Work balancing	Adoption system: Activity integration
Adaption, reallocation of resources, flexible resource basket, challenging status quo Senior management support and commitment, favourable atmosphere, empowering subordinates	6	Organisational flexibility Senior management support	Health system characteristics: organisational support
Quality control, monitoring, external partners, supporting local facilitators, promoting learning, regular monitoring, developing local capacity, partnerships	7	Promoting learning Quality control Developing local capacity and linkages	Broader context: External monitoring

## Results

**Fig. 1:**
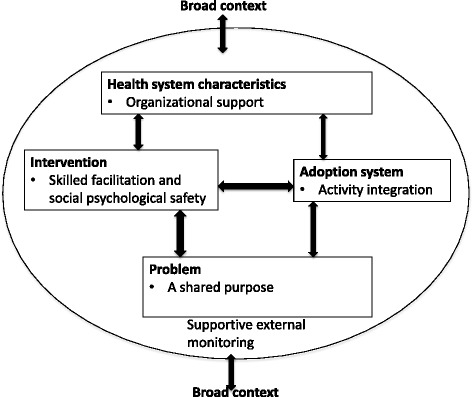
The elements for harnessing PAR to strengthen health managers’ capacity. This is an illustration of the complex bi-directional and intricate interactions between the different elements for harnessing PAR to strengthen health managers’ capacity

The elements for harnessing PAR to strengthen health management were synthesised into five domains with bi-directional relations in accordance to the Atun et al. [37] framework (Fig. 1). For the problem domain, we found that, for PAR to be harnessed, the issues under investigation need to have been arrived at in a shared manner between the managers and the researchers or external parties. In the intervention domain, skilled facilitation and social psychological safety aspects of the intervention were found. Activity integration into organisational procedures was noted in the adoption domain. Under the health system characteristics domain, organisation support was found to be essential. Finally, in the broader context, supportive external monitoring provided by external parties or researchers played an important role. These five domains interacted with each other in several directions and in non-linear ways (Fig. 1). Each of these five domains is explored in greater detail below.

### The problem: a shared purpose

A shared purpose was found to be central in defining the problem to be tackled when using PAR to strengthen health managers’ capacity [18, 19, 35, 38–41]. This was depicted as having a shared purpose and motivation towards strengthening managers’ capacity among the parties involved and a focus on individual managers’ development.

While PAR processes inherently trigger a sense of shared purpose, an overt shared goal from the onset shaped managers’ ability to harness the learning opportunities offered by PAR [38, 41]. This enabled the managers to determine and define their management skills gaps. A common drive fostered the development of clear and manageable goals for developing specific skills among the managers [38, 39]. Without this shared purpose, a polarised pursuit of different ends can emerge due to low motivation levels and misunderstandings. This disrupted the development of specific management skills because key phases and tenets of PAR were missed. For example, in one of the papers reviewed, it was found that, during the PAR processes, the health managers were more preoccupied with recording specific project outcomes, and yet the external partners intended the review phase of PAR to enable a deeper reflection on actions in order to facilitate development of critical thinking skills [19].

A focus on the development of individual managers’ abilities bolstered the attainment of a shared purpose [18, 39]. An explicit definition of individual managers’ needs and making efforts to meet them motivated health managers to actively take part in the PAR learning processes. In addition, paying attention to their explicit expectations and motivations created a sense of being supported to achieve personal goals [41]. As a result, enthusiasm and commitment to undertake the PAR process was garnered, which supported the learning processes that ensued [39, 41].

### The intervention: skilled facilitation and social psychological safety

In the intervention domain, skilled facilitation of the PAR process and a sense of social psychological safety enabled the harnessing of PAR [18, 19, 34, 35, 38–41]. In this review, we found that social psychological safety is a consequence of skilled facilitation although we discuss them separately to provide adequate detail to both.

#### Skilled facilitation

Skilled facilitation, which meant the handling of the PAR process with special skills that promoted provocative and creative ways of thinking as opposed to a ‘business as usual’ attitude, facilitated the garnering of opportunities to strengthen managers’ capabilities [18, 19, 34, 35, 38–41]. Skilled facilitation involved skills for effective management of learning groups, enabling self-efficacy, tailored facilitation and promotion of reflective thinking [19, 34, 35, 38–41]***.***

Effective management of learning groups entailed preparing for the meetings, having a manageable group size, choosing an appropriate setting for the meetings (time, sitting arrangements and venue), ensuring a good level of participation by all participants and managing the feedback process [18, 34]. Meeting preparations required a mastery of the PAR principles and the cycles of learning. In terms of size, our review indicated that smaller learning sets are easier to manage. Typically, a group of between 7 and 12 participants is encouraged to allow a good level of participation, group heterogeneity and support [34, 35, 41]. Choosing the appropriate setting and timing for the meetings required prior inquiry about the most appropriate setting and timing from the participants, supported by advance scheduling [34, 41]. Wrong timing led to less concentration and contributed to distractions [38, 39]. Similarly, an appropriate venue with minimum distractors and conducive for open sharing enhanced learning. Allowing long and sustained engagements in the learning process so as to develop the needed capabilities was the other important aspect of timing [39, 42]. Circular sitting arrangements during meetings were noted to maximise interactions, promoting openness, familiarity and minimising negative power dynamics [18, 34]. Importantly, ensuring a good level of engagement of all participants was found to be critical. This was attained by consciously allocating specific time to every participant and actively encouraging airing out of individual experiences and thoughts to avoid a ‘group think’ situation [34, 39].

Facilitation that triggered self-efficacy among the participants enabled learning. Triggering self-efficacy in a PAR process required the demystification of ‘inability myths’ among participants [35]. This required promotion of participation, open learning [19, 35] and building of trust among the participants [41]. Demystification was best done in the initial meetings, in order to create an atmosphere of hope and determination to improve [35]. This was often achieved through a well thought through charismatic and inspiring introductory session, as noted in a study undertaken in Tanzania to improve managers’ capacity at district level [35].

Tailored facilitation consisted of adapting to the needs of the participants and their specific contexts [34, 35, 40, 41], whilst being open minded and sensitive to the different needs of the participants. It created a sense of responsiveness and motivated the participants to engage more with the process, hence garnering all possible skills. For example, rotating meetings among the different organisations or departments represented by PAR participants was appreciated in one of the studies conducted in South Africa [41]. It created a sense of local relevance and was also viewed as a cost saving mechanism for those who hosted the meetings. This re-emphasised the need to have a shared purpose as described above.

A facilitator should be able to create an atmosphere that promotes reflective thinking, a process by which participants deeply meditate upon their actions to draw lessons, promoting identification of gaps and enhancing skill-building; overall, this nurtured goal re-orientation and creativity among managers [19, 38, 43]. To promote reflective thinking, an atmosphere that encouraged asking questions, following up on action points, and challenging and being challenged was cultivated [19, 38, 41]. Approaches to promoting reflective thinking included having enough time for reflection for each participant during meetings, allowing adequate time between meetings and encouraging open discussions [19]. In addition, record keeping was found to be a good basis upon which reflection was hinged, serving as a memory of past events [18]. It triggered discussions around why certain actions were taken and why they succeeded or failed, and allowed for a broad critical reflection. However, the purpose and methods of record keeping had to be commonly understood and appreciated for it to be an effective refection and learning tool, otherwise it would be reduced to a shallow record of events [19, 38, 43]. For example, in a study undertaken in Ireland, indicating learning areas prior to a learning session helped members follow-up on learning outcomes [19]. To undertake reflective thinking, participants found it easier to record oral reflections rather than to produce structured and written documentation [38, 41, 43]. This was so because the PAR learning processes were largely interactive, which made simultaneous note-taking burdensome. Additionally, the culture of recording reflections was relatively new in the study conducted in Uganda, Tanzania and Ghana [38].

#### Social and psychological safety and support

The second attribute of the intervention was the social and psychological safety and support, specifically the security that individual participants of the PAR learning process experienced when engaging in a learning group. This was facilitated by four main attributes, namely a non-threatening environment, confidentiality, a heterogeneous learning group and social capital. As noted earlier, these attributes are both a direct and indirect consequence of skilled facilitation. These together created a conducive learning atmosphere characterised by free and supportive interactions [18, 19, 38–41, 43].

Managers in the reviewed studies described a non-threatening environment as one which fostered open and free communication among persons without the fear of causing damage to existing relationships [38, 40, 43]. Interactions involved both vertical and horizontal relations. Therefore, securing these relations in a space of free and open interactions was paramount if individual managers were to effectively harness opportunities to improve their management competencies. Furthermore, a non-threatening environment allowed for a non-judgmental atmosphere where people expressed themselves freely [40, 43]. Such ‘safe’ spaces of interactions created a positive mental state of learning, which consequently affected their abilities to learn.

To achieve this, the interactions within the learning groups were confidential [41]. Members of a learning group felt mentally safe to disclose their weaknesses and to discuss different challenges without any risks of unconsented sharing of information, as emphasised in two studies from Australia and USA among healthcare managers [18, 43]. As an initial step towards confidentiality, some of the studies ensured participant heterogeneity within the PAR learning groups. For example, members of each learning group were from different organisations or departments [19, 40, 41, 43].

A heterogeneous learning group also meant the involvement of stakeholders that had different power relations, which functioned as a facilitator for triggering the needed changes within an organisation at different levels [34]. A heterogeneous group could be achieved through having representation from several units of the organisation, which was also vital in ensuring organisational level buy in and support. This type of support, as shall be seen under the health system characteristics, is essential for facilitating learning by doing [40]. It also provided a proper group mix of talents or professionals in order to complement each other’s abilities and thereby strengthen overall managers’ capacity [19, 40]. Such heterogeneity was useful for collaborations among participants and further built social capital by creating interdependency through professional networks [39, 43].

Social capital was found to be a consequence of the mental safety among participants of a PAR learning process as noted above [34, 39]. It is also developed through a process of interaction among participants aided by skilled facilitation [34, 43]. Within a learning group, participants undertook collective efforts towards developing each other’s capabilities, which created a sense of mutual dependency. This built trust and motivated participants to strive for more as a gesture of fitting within the learning groups. In addition, learning spaces were expanded as participants worked together to accomplish given tasks. Further, social capital was vital for networks of consultations, which created “professional safety nets” as noted in studies undertaken in Ireland and South Africa [19, 41].

### The adoption system: activity integration into organisational procedures

In the adoption system, we focus on the receptiveness of the health managers and their organisations to the PAR approach. As the ‘problem’ and the ‘intervention’ influence the managers’ interest in the PAR process, so does integration into organisational procedures. To integrate, two attributes were found in the literature reviewed, the existence of local champions and mainstreaming of activities within organisations [18, 35, 38–40].

Local champions of the PAR process were the coordinators of the entire process within their local organisations. They played the roles of ensuring regular attendance of meetings, supporting action taking, overseeing quality control processes and documenting key outcomes [38]. Championing was an inbuilt mechanism of ensuring continuity of the PAR learning processes [35]. Local champions had a working knowledge and experience of facilitating the PAR learning process, a skill and experience built through an on-going process of working closely with external facilitators [38]. To ensure the effectiveness of local champions, they needed to have long-term stability within the organisation; otherwise, regular transfers or high staff turnover would render such investments futile or less effective [19, 35].

Secondly, the mainstreaming of PAR activities was found to trigger a sense of ownership of the process as well as the outcomes within the organisation, which facilitated the development of a shared purpose [38]. To mainstream PAR, attention was paid to the operational aspects of the organisations where the participants belonged and their usual work load, organisational budgets and needs [38, 39]. For example, over-loaded managers eventually became ineffective and failed to learn intended lessons from the PAR process. Documentation requirements were the main challenge to busy District Health Management Team members, as noted in a study undertaken in Ghana, Uganda and Tanzania [38], wherein the use of diaries to aid the process of reflection was decided upon and promoted by the external partners. While the local teams adopted them, their full acceptance and use was highly variable. The managers battled with a lack of clarity in the documentation templates or processes, competing demands within their organisations, interruptions from higher authorities and other parallel projects. It was therefore recommended that workload balancing be openly discussed between parties.

### The health system characteristics: organisational support

Organisational flexibility and senior management support were noted under the organisation support domain [19, 34, 35, 38, 39, 43]. Organisational support interacted with intervention attributes and the adoption system to harness the PAR opportunities to strengthen managers capacity.

Organisational flexibility meant having opportunities to reallocate organisational resources and to challenge the status quo for positive changes [35, 39]. Flexibility enabled managers to redistribute resources to support the learning process or action plans and to practice acquired skills [35]. As such, flexibility enabled the development of self-efficacy and empowered managers as it widened their space of operation and decision-making [18, 39]. Managers needed a flexible resource basket within which they could make reallocations in line with learning outcomes from the PAR process. These included both financial and non-financial resources coupled with the possibility for managers to create more resources as noted by Doyle in her study of developing leaders in a healthcare context [19]. Challenging the status quo, on the other hand, entailed the modification of policies, regulations and strategies that were non-progressive [39].

Secondly, having the backing from senior management [34, 35] was manifested in their commitment to strengthen managers’ capacity through the allocation of resources but also the creation of a favourable atmosphere [43]. Support from senior managers and commitment by organisational leaders acted as a motivating factor for managers to implement action points arising from the PAR process [35]. PAR typically required a highly flexible environment of free and open interactions to facilitate learning. Senior management support was demonstrated through involvement in the PAR meetings, follow-up on progress, commitment of resources to the process and empowering of managers to undertake specific tasks aimed at strengthening their capacity [18, 35].

### The broader context: supportive monitoring

The broader context within which PAR was implemented played an important role in influencing how its opportunities were exploited to strengthen managers’ capacity. From the literature reviewed, we identified supportive monitoring as forming the broader context [18, 19, 35, 38, 41, 43].

Supportive monitoring was a form of quality control for the learning process. The use of PAR as a learning tool usually occurred in partnerships involving parties within the organisation (in this case, health managers) and those without (researchers, partners, experts or senior management). Supportive monitoring was understood as the role of external players, such as researchers or partners, in the PAR process [34, 38, 41].

External monitoring took the form of attending scheduled review meetings, monitoring and supporting the interaction processes to ensure free and open interactions and supportively being engaged with group learning discussions [19, 41]; this facilitated overt identification of successes, challenges and possible solutions [34, 38, 41]. In addition, the external monitoring team played an important role in providing support to local teams in the process of reflection and learning [38, 41]. As noted earlier, skilled facilitation was critical in ensuring reflective learning [38]. External monitoring therefore supported the process of building the capacity of local champions to facilitate the PAR learning process.

Regular external monitoring also facilitated the building of relationships between partners [38, 43]. Such relations were the basis for continued networking, which was essential for continuous capacity-building synonymous with the PAR approach [41]. Face-to-face interactions with external teams were found to be good at advancing networking relations because they helped develop trust and confidence among the partners involved as noted by Doyle [19], Leggat et al. [18], Mshelia et al. [38] and Roberts [43] in their studies reviewed herein. These positive relations formed the basis upon which managers’ responsiveness to feedback from external partners was built [38, 43]. Nonetheless, other forms of monitoring, such as email interactions and phone calls between external and local partners, were also found to be useful ways of sharing feedback and monitoring the implementation of PAR activities [38, 43].

## Discussion

In the discussion, we reflect on the interrelationships between the elements identified for harnessing PAR to strengthen health managers’ capacity. In addition, we reflect on a wider application of these elements to health systems strengthening and implementation research.

As noted in the results section, the five elements identified interacted with each other in bi-directional ways in accordance with the framework adopted for this synthesis. This intricate relationship reflected the non-linear but complex nature of health systems in general and managers’ capacity development in particular [15, 26]. For example, a shared purpose was developed out of a full engagement of participants, which was an attribute of skilled facilitation. On the other hand, having a shared purpose created a sense of social psychological safety by nurturing the trust among participants and facilitated full involvement [43].

Under skilled facilitation, we found that reflection was a critical part of the PAR process because it allowed the development of critical thinking skills. Tackling health systems issues involves more than reacting to problems; instead, engaging in deep and broad reflection on actions helps to advance systems strengthening [44]. PAR offers an opportunity to facilitate such deep and broadened reflection on action through its reflective spaces, which are typically inclusive and engaging [45, 46].

In addition to fostering ingenuity and resourcefulness of all stakeholders involved, PAR strengthens health systems by promoting safe spaces of interaction [47]. The inviolability of such spaces is eminent, especially when undertaking implementation research projects that seek to answer the ‘how and why’ questions of strengthening health systems. According to our review, there was an overt necessity for external partners or researchers to consciously promote local inclusion and engagement in order to strengthen managers’ capacity. Nonetheless, such engagement is often a challenge for two interrelated reasons, one is a lack of experience in facilitation and the ‘experts syndrome’, these obstacles therefore need to be reflected upon and dealt with accordingly in order to strengthen managers’ capacity specifically and health systems as a whole [48, 49].

Activity integration into usual organisational operations was an integral part of this intricate relationship of the elements for harnessing PAR to strengthen managers’ capacity. Mainstreaming is a well-known means of vertical scale-up and promoting local relevance of interventions [50]. Ensuring that the PAR processes and their outcomes are compatible with the goals and values of the health institutions is therefore critical. Compatibility reduces possible conflict between actors, structures and procedures. In addition, compatibility facilitates uptake of the outcomes of the new health interventions resulting from the PAR processes and a reduction of effort duplication [51]. Enhancing compatibility requires a systems thinking approach. Indeed, PAR, through its principles of collaborative resource, plural structures and dialogue, promotes systems thinking [16, 42, 52, 53].

The benefits of integration withstanding, it is common to find initiatives aimed at strengthening managers capacity that are planned and implemented parallel to the existing local systems, especially in low-income countries [48, 49]. Consequently, the scale-up of successful interventions is often a challenge among these countries due to weakened systems [48, 49, 54]. This is further compounded by rigid hierarchical structures that create a shrunk decision-making space and make it difficult to act upon new insights [55, 56]. Approaches such as PAR develop local capacity by actively involving the local stakeholders in the implementation of interventions. This kind of stakeholder engagement aids in the development of self-efficacy − the belief in one’s ability to undertake a given task. Self-efficacy is hinged on organisational flexibility to expand health managers’ decision-making spaces. In the literature reviewed, a positive self-image of the participants played a critical role in developing their management capacity [18]. In addition, as noted earlier, the PAR process should be viewed as adding value to both the health managers’ competencies and the wider organisation, which demonstrates the relationship between integration and organisational support domains.

Similarly, the organisational support interacted with the adoption system in bi-directional ways. For example, with mainstreaming, organisational support was garnered on the one hand. On the other, organisational support is essential for the mainstreaming of interventions into existing health system structures. Organisational support acts as a bridge between testing and implementing proven interventions [57]. Securing this kind of support through early and continued engagements lays a good foundation for creating lasting changes aimed at strengthening health systems. However, the temptation to override existing systems is often high given the complex nature of systems and some of the inherent weaknesses, such as weak management capacity, that could take much longer to deal with [58, 59]. Nonetheless, bypassing existing organisational structures to create faster and more controlled pathways could be counterproductive in the long run.

Parallel initiatives continue to perpetuate the weakness of local health systems, especially where the negotiation space is often skewed in favour of external stakeholders [60]. While such parallel structures provide needed services by complementing the existing structures, they often drain and disrupt existing human and other resources; moreover, they are usually short lived [54, 60]. At the end of their services, the enduring systems are usually weaker. PAR principles, such as engagement of local stakeholders and having a shared purpose, play an important role in leveraging the different strong points of both local and external stakeholders in systems strengthening. The continued interaction of the health system with external parties’ aids in strengthening it in many ways, including the development of professional support networks or social capital, offering monitoring and quality control support, capacity development, and actual temporary resource support [57, 61].

### Study limitations

The exclusion of grey literature represents a study limitation as this could have enriched or even diversified the synthesis given the limited published literature of the subject matter. However, we believe that the published literature offered a fair representation of what was intended as the study aim.

## Conclusions

The use of PAR to strengthen managers’ capacity should be done in consideration of the elements identified and discussed. These elements intricately interact to allow the successful harnessing of PAR. Additionally, although these elements cut across all contexts, further contextualisation of the specific elements needs to be undertaken specifically because contextualisation is synonymous with PAR principles that typically advocate for local relevance. The use of PAR for health interventions is therefore appropriate for health systems interventions given the complexity of the health system. Furthermore, PAR has the potential to create and nurture an environment of trust and frank collaboration among stakeholders so as to unveil underlying conditions, mechanisms and pathways for systems strengthening.

To reflect on the quality of the synthesis undertaken, we undertook a self-assessment of the synthesis based on the Confidence in the Evidence from Reviews of Qualitative Research (CERQual) guidelines. We looked at the methodologic quality, relevance, coherence and adequacy of data [62]. This revealed that the CIS method was indeed appropriate for this kind of review and its application is carefully detailed in the methods section. We thought that the relevance of the evidence is high given the lack of such collated knowledge on harnessing PAR at a time when such approaches are being promoted. In terms of coherence, the findings revealed a pattern that is confirmed across individual studies, while the adequacy of data may be limited due to minimal studies published in the area. Studies from future primary research could add value to this review. In addition, an independent review of the synthesis based on CERqual could even be more appropriate. Nonetheless, we postulate that, while the findings from this review can be taken with moderate confidence due to the possibility of inadequate data, it does provide a solid base for applying PAR to health systems strengthening initiatives.
